# Recombinant collagenase from *Grimontia hollisae* as a tissue dissociation enzyme for isolating primary cells

**DOI:** 10.1038/s41598-020-60802-z

**Published:** 2020-03-03

**Authors:** Keisuke Tanaka, Teru Okitsu, Naoko Teramura, Katsumasa Iijima, Osamu Hayashida, Hiroki Teramae, Shunji Hattori

**Affiliations:** 1 Nippi Research Institute of Biomatrix, Toride, Ibaraki 302-0017 Japan; 2 Institute of Industrial Science, The University of Tokyo, Meguro, Tokyo 153-8904 Japan; 3 Faculty of Teacher Education, Shumei University, Yachiyo, Chiba 276-0003 Japan

**Keywords:** Regenerative medicine, Applied microbiology

## Abstract

Collagenase products are crucial to isolate primary cells in basic research and clinical therapies, where their stability in collagenolytic activity is required. However, currently standard collagenase products from *Clostridium histolyticum* lack such stability. Previously, we produced a recombinant 74-kDa collagenase from *Grimontia hollisae*, which spontaneously became truncated to **~**60 kDa and possessed no stability. In this study, to generate *G. hollisae* collagenase useful as a collagenase product, we designed recombinant 62-kDa collagenase consisting only of the catalytic domain, which exhibits high production efficiency. We demonstrated that this recombinant collagenase is stable and active under physiological conditions. Moreover, it possesses higher specific activity against collagen and cleaves a wider variety of collagens than a standard collagenase product from *C. histolyticum*. Furthermore, it dissociated murine pancreata by digesting the collagens within the pancreata in a dose-dependent manner, and this dissociation facilitated isolation of pancreatic islets with masses and numbers comparable to those isolated using the standard collagenase from *C. histolyticum*. Implantation of these isolated islets into five diabetic mice led to normalisation of the blood glucose concentrations of all the recipients. These findings suggest that recombinant 62-kDa collagenase from *G. hollisae* can be used as a collagenase product to isolate primary cells.

## Introduction

Collagenase has been widely used to isolate a variety of specialised cell types from attendant connective tissue where collagen is a major component. The isolation of primary cells is necessary to study cell function in basic research^1,2^; isolation of cancer stem cells of solid organs is also important for the investigation of cancer pathophysiology in clinical research^3,4^. Moreover, techniques for isolation of primary cells are essential aspects of therapeutic procedures in the fields of transplantation^5,6^ and regenerative medicine^7,8^. Collagenase products commonly used for these purposes are derived from *Clostridium histolyticum*. Clostridial collagenase products are known to exhibit lot-to-lot and intra-lot variability even when collagenase is highly purified, resulting in variable isolation outcomes^9–12^; thus, there is room to improve these collagenase products.

Clostridial collagenase products contain two components: class I (ColG) and class II (ColH) collagenases. These components play different roles in collagen digestion^13,14^ and both are necessary for efficient isolation of primary cells^15^. The composition of the enzyme blend can be modified to achieve optimisation for specific protocols and organ characteristics^16^. However, the combination of these two components in a single enzyme product impairs its homogeneity and might induce an auto-degradation process, leading to lot-to-lot and even intra-lot variability in clostridial collagenase products^9^.

*Grimontia hollisae* is a Gram-negative bacterium that was previously classified in the genus Vibrio^17^; several Vibrio species are known to produce collagenases^18^. A collagenase from *G. hollisae* strain 1706B has previously been purified and characterised as a single **~**60-kDa protein possessing high collagenolytic activity^19^. This collagenase enzyme is stable and most active at physiological pH and temperature. Moreover, this enzyme is even able to degrade tanned leather containing many types of collagens that are much more tightly and densely cross-linked with each other, compared with collagens in native skin tissue^20,21^. These properties imply that the ~60-kDa protein from *Grimontia hollisae* strain 1706B could isolate primary cells as a single-component collagenase product.

Recombinant collagenase from *G. hollisae* strain 1706B has been successfully produced using the *Brevibacillus* Expression System^22,23^. During cloning of the gene encoding the *G. hollisae* collagenase, we previously found that the ~60-kDa collagenase secreted by the bacterium is initially translated as a 74-kDa protein. Following expression of the 74-kDa recombinant protein, most of the collagenase proteins are spontaneously truncated to the ~60-kDa form; importantly, the 74-kDa and ~60-kDa proteins exhibit distinct collagenase activities^23,24^. Therefore, the 74-kDa recombinant protein is unsuitable for establishing a collagenase product because the product would lack homogeneity due to the presence of two components; moreover, the product would lack stability because of the uncontrollable spontaneous truncation mechanism. Thus, the 74-kDa recombinant protein does not exhibit the usual features that the recombinant protein is superior to the native protein in terms of homogeneity and stability^25^. To generate a recombinant collagenase from *G. hollisae* to establish an enzyme product for isolating primary cells, direct expression of the ~60-kDa recombinant protein is needed. However, efforts to achieve this would not be promising because it is difficult to reliably predict the activity and quantity of the resulting recombinant protein, despite recent advancements in recombinant protein expression technology.

The aim of this paper is twofold. First, we design the recombinant ~60-kDa collagenase from G. *hollisae* and test whether it can be directly expressed using the *Brevibacillus* Expression System. Second, we test whether the ~60-kDa recombinant protein possesses collagenolytic activity and stability sufficient to establish a collagenase product, as well as whether it can be used to isolate primary cells. To evaluate the potency of the recombinant protein to isolate primary cells, we adopt isolation of mouse pancreatic islets because a system for assaying the morphology and function of pancreatic islets has already been established^26^; moreover, collagenase products for islet isolation have been more extensively developed in clinical settings, compared with collagenase products for any other primary cell isolation procedures^27^.

## Results

### Design and expression of truncated ~60-kDa recombinant proteins from *G. hollisae* for assessment of the collagenolytic activity

**Figure 1:**
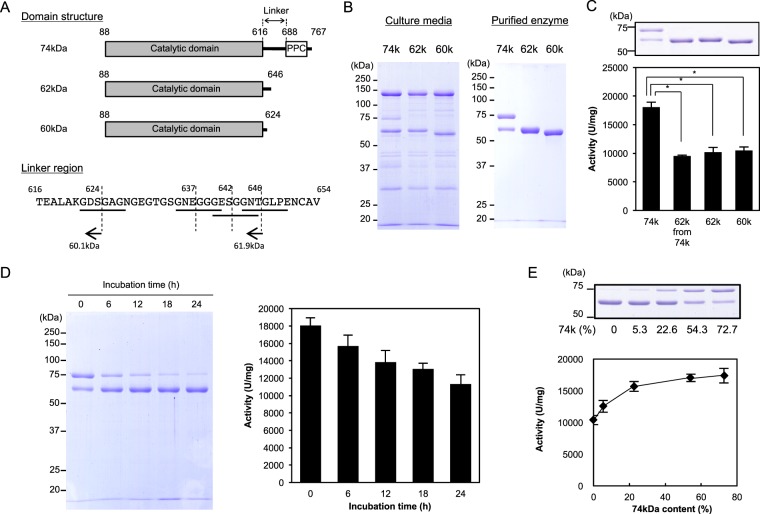
Graphical representation of recombinant *Grimontia hollisae* collagenases and their collagenolytic activities. (**A**) Recombinant proteins were designed as 74 kDa (aa 88–767, including the PPC domain), 62 kDa (aa 88–646), and 60 kDa (aa 88–624) of *G. hollisae* collagenase. The amino acid sequence of the linker region is highlighted and four G-X-Y repeats are underlined. (**B**) Recombinant collagenases were purified from *Brevibacillus* culture medium by diethylaminoethanol-Sepharose chromatography. Ten microliters of culture media (left panel) and two micrograms of purified recombinant collagenases (right panel) were analysed by sodium dodecyl sulfate polyacrylamide gel electrophoresis (SDS-PAGE) using a 7.5% polyacrylamide gel. Numbers on the left are molecular masses (in kDa) of the markers. (**C**) Collagenolytic activities of recombinant collagenases were determined using FITC-collagen. Values represent the average of triplicate trials ± standard deviation. *P < 0.01, determined by one-way analysis of variance. The uncropped gel is included in a Supplementary Information File. (**D**) Stability analysis of recombinant 74-kDa collagenase. Recombinant collagenase was incubated at 37 °C in 50 mM Bis-Tris-HCl buffer (pH 7.5) containing 0.2 M NaCl and 5 mM CaCl_2_. After incubation for various time intervals, the reaction mixture was analysed by SDS-PAGE (left panel) and its collagenolytic activity was measured using FITC-labelled type I collagen (right panel). Values represent the average of triplicate trials ± standard deviation. (**E**) Collagenolytic activities of recombinant collagenases with different proportions of 74-kDa and 62-kDa proteins were determined using FITC-collagen. Values represent the average of triplicate trials ± standard deviation. The uncropped gel is included in a Supplementary Information File.

To design recombinant proteins identical to the truncated ~60-kDa protein that is spontaneously generated from the recombinant 74-kDa collagenase from *G. hollisae*, we analysed the linker region (~6.9 kDa) of the 74-kDa protein between its catalytic domain (~59.2 kDa) and the C-terminal region (~8.5 kDa) that contains the pre-peptidase C-terminal (PPC) domain. We found that the linker region contains four sites that are presumably susceptible to cleavage by collagenase; these sites contain collagenous sequences of G-X-Y repeats, where G represents glycine, while both X and Y represent any amino acids (Fig. 1A). Based upon this finding, we designed 60-kDa and 62-kDa proteins possessing the shortest and longest linker lengths, respectively. We successfully expressed these proteins using the *Brevibacillus* Expression System on the millilitre scale, then purified them using chromatography (Fig. 1B). When we compared these purified proteins with the recombinant 74-kDa collagenase by sodium dodecyl sulfate polyacrylamide gel electrophoresis (SDS-PAGE) analysis, we found that the spontaneously truncated ~60-kDa protein from the recombinant 74-kDa collagenase was 62 kDa, rather than 60 kDa (Fig. 1B). In addition, we found that the same types of protein molecules were present in both the spontaneously truncated 62-kDa protein and the recombinant 62-kDa protein, based on assessment of their C-terminal amino acid sequences using quadrupole time-of-flight mass spectrometry (Fig. 1A and S1). Moreover, based on the outcomes of three independent collagenolytic assays using fluorescein isothiocyanate (FITC)-labelled collagen (Fig. 1C), we determined that both 62-kDa and 60-kDa recombinant proteins possessed comparable degrees of collagenolytic activity (10,203 ± 828 U/mg vs 10,495 ± 612 U/mg, p = 0.61, one-way analysis of variance [ANOVA]). The activities of the 62-kDa and 60-kDa recombinant proteins were comparable to the activity of the spontaneously truncated 62-kDa protein (10,203 ± 828 U/mg and 10,495 ± 612 U/mg vs 9,531 ± 152 U/mg, p > 0.10, one-way ANOVA), but these activities were significantly lower than the activity of the recombinant 74-kDa collagenase (10,203 ± 828 U/mg and 10,495 ± 612 U/mg vs 18,077 ± 867 U/mg, p < 0.01, one-way ANOVA). We then performed stability analysis of the recombinant 74-kDa collagenase and observed that it gradually converted to the 62-kDa form over time (Fig. 1D). We also found that the collagenolytic activity was not directly proportional to the ratio of 74-kDa and 62-kDa proteins in a blended preparation (Fig. 1E). Based on these results, we concluded that the recombinant 74-kDa collagenase exhibits structural instability and inconsistent collagenolytic activity; thus, we determined that the 74-kDa protein should be excluded from further preparations to produce a stable recombinant collagenase product.

### Fundamental characterisation of recombinant 62-kDa collagenase from *G. hollisae*

**Figure 2:**
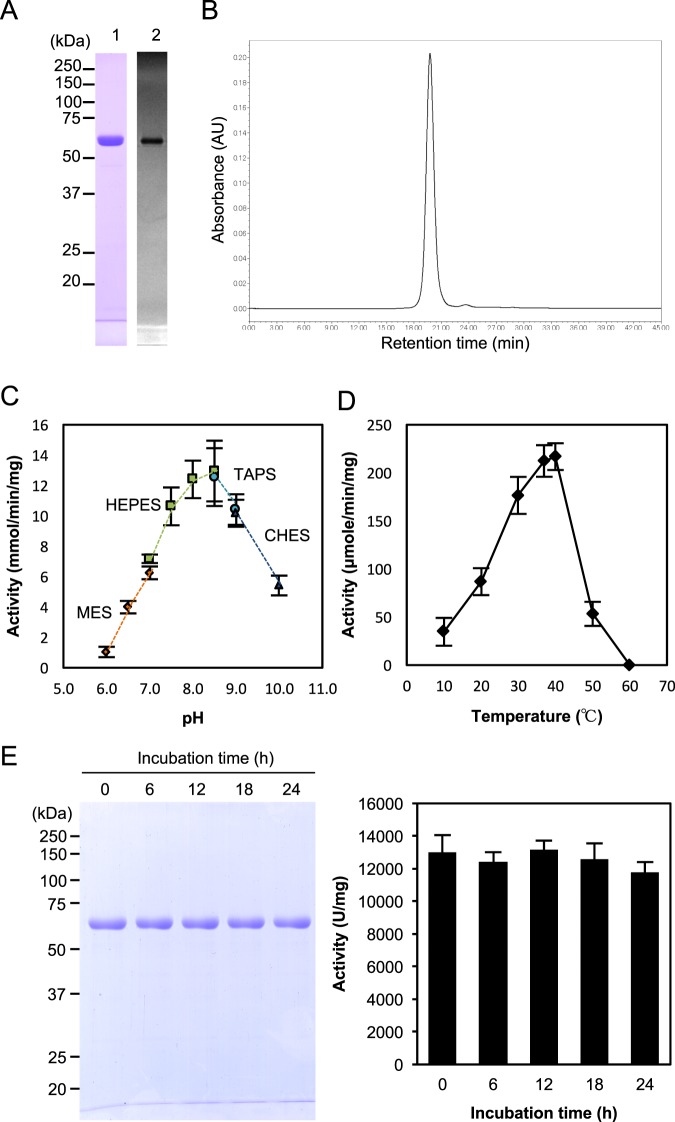
Characterisation of recombinant 62-kDa collagenase from *Grimontia hollisae*. (**A**) Purified recombinant 62-kDa collagenase was analysed by sodium dodecyl sulfate polyacrylamide gel electrophoresis (SDS-PAGE) (lane 1) and real-time gelatin zymography (lane 2). Numbers on the left are molecular masses (in kDa) of the markers. (**B**) Size exclusion chromatogram of recombinant 62-kDa collagenase. Size exclusion chromatography was performed on an Alliance 2895 system using a Superdex 200 HR10/30 column. The sample was loaded onto a column and eluted in an isocratic manner with 50 mM Bis-Tris-HCl (pH 7.5) containing 0.2 M NaCl, at a flow rate of 0.75 ml/minute. The separated protein fraction was detected at 220 nm. (**C**) pH-dependence of recombinant 62-kDa collagenase. Collagenase and FALGPA were mixed in each buffer mentioned below, then incubated at 30 °C for 5 minutes. The following buffers were used: 50 mM MES (pH 6.0–7.0), 50 mM HEPES (pH 7.0–8.5), 50 mM TAPS (pH 8.5 and 9.0), and 50 mM CHES (pH 9.0 and 10.0) containing 0.2 M NaCl and 5 mM CaCl_2_. (**D**) Temperature-dependence of recombinant 62-kDa collagenase. Collagenase was incubated with the Pz peptide in 50 mM HEPES (pH 7.5) containing 0.2 M NaCl and 5 mM CaCl_2_ at various temperatures (10–60 °C). (**E**) Stability analysis of recombinant 62-kDa collagenase. Recombinant collagenase was incubated at 37 °C in 50 mM Bis-Tris-HCl buffer (pH 7.5) containing 0.2 M NaCl and 5 mM CaCl_2_. After incubation for various time intervals, the reaction mixture was analysed by SDS-PAGE (left panel) and its collagenolytic activity was measured using FITC-labelled type I collagen (right panel). Values represent the average of triplicate trials ± standard deviation.

For further evaluation of recombinant collagenase from *G. hollisae*, we decided to adopt the 62-kDa protein rather than the 60-kDa protein, based upon our experience that stable transformants carrying the construct for 62-kDa protein were able to be obtained, while those for 60-kDa protein were not. We used the stable transformants to express the 62-kDa recombinant protein in the *Brevibacillus* Expression System on the litre scale, then purified the expressed proteins. When we evaluated the recombinant protein by SDS-PAGE, we found that this protein was present as a single band at 62 kDa (lane 1 in Fig. 2A); using size exclusion chromatography, we found that this protein existed in monomeric form without aggregation (Fig. 2B). Using gelatin zymography, we determined that the 62-kDa protein possessed gelatinolytic activity (lane 2 in Fig. 2A). Subsequently, using synthetic substrates for analysis of collagenolytic activity, we found that the 62-kDa recombinant protein exhibited optimal activity in the pH range of 7.5–9.0 (Fig. 2C), and in the temperature range of 30–40 °C (Fig. 2D). Moreover, we analysed the stability of the 62-kDa recombinant protein and observed that it remained intact without degradation for up to 24 hours at 37 °C; during the 24-hour incubation period, it retained stable collagenolytic activity (Fig. 2E). Based on these results, we concluded that the recombinant 62-kDa collagenase from *G. hollisae* exhibits structural stability and consistent collagenolytic activity; thus, we determined that this recombinant protein is suitable for use in a collagenase product.

### Kinetic analysis of recombinant 62-kDa collagenase from *G. hollisae* by comparison with purified collagenase product from *C. histolyticum*

To determine specific activity and enzyme kinetic parameters (i.e., K_m_, V_max_, and K_cat_), we subjected the recombinant 62-kDa collagenase from *G. hollisae* to collagenolytic assays using two types of substrates, FITC-labelled collagen and a synthetic peptide of furylacryloyl-Leu-Gly-Pro-Ala (FALGPA); thereafter, we compared the outcome with those of purified collagenase product from *C. histolyticum* (Table 1). We found that the specific activity of the recombinant *G. hollisae* collagenase was more than three-fold higher than that of the purified collagenase product from *C. histolyticum* against both substrates (5,490 U/mg vs 1,766 U/mg for FITC-collagen; 9.39 U/mg vs 2.60 U/mg for FALGPA). Moreover, whereas the K_m_ of the recombinant *G. hollisae* collagenase was comparable with that of the purified collagenase product from *C. histolyticum* (1.09 ± 0.35 × 10^−3^ mM vs 1.89 ± 0.42 × 10^−3^ mM for FITC-collagen, p  =  0.07; 2.28 ± 0.23 mM vs 2.03 ± 0.48 mM for FALGPA, p  =  0.47), the V_max_ and K_cat_ of recombinant *G. hollisae* collagenase were both higher than the V_max_ and K_cat_ of the purified collagenase product from *C. histolyticum* ([V_max_: 4.06 ± 0.84 × 10^−4^ mM/s vs 1.61 ± 0.39 × 10^−4^ mM/s for FITC-collagen, p = 0.01; 0.61 ± 0.13 mM/s vs 0.19 ± 0.03 mM/s, for FALGPA, p < 0.01], [K_cat_: 25.14 ± 5.20 s^−1^ vs 18.69 ± 4.46 s^−1^ for FITC-collagen, p = 0.18; 37.53 ± 8.03 s^−1^ vs 22.15 ± 2.94 s^−1^ for FALGPA, p = 0.04]). Based on these findings, we determined that the recombinant 62-kDa collagenase from *G. hollisae* is comparable with the purified collagenase product from *C. histolyticum* in terms of its affinity for collagen; furthermore, the recombinant 62-kDa collagenase from *G. hollisae* is comparable with or superior to the purified collagenase product from *C. histolyticum* in terms of its ability to catalyse the cleavage of collagen.

**Table 1:** Kinetic constants of recombinant *G. hollisae* and purified *C. histolyticum* collagenases.

Substrate	Enzyme strain	Sp act (U/mg)	Mean + SD		
			K_m_ (mM)	V_max_ (mM/s)	K_cat_ (s^−1^)
FITC-collagen	*G. hollisae*	5,490	(1.09 ± 0.35) × 10^−3^	(4.06 ± 0.84) × 10^−4^**	25.14 ± 5.20
	*C. histolyticum*	1,766	(1.89 ± 0.42) × 10^−3^	(1.61 ± 0.39) × 10^−4^	18.69 ± 4.46
FALGPA	*G. hollisae*	9.39	2.28 ± 0.23	0.61 ± 0.13*	37.53 ± 8.03**
	*C. histolyticum*	2.60	2.03 ± 0.48	0.19 ± 0.03	22.15 ± 2.94

The activities of *G. hollisae* and *C. histolyticum* collagenase were determined using FITC labeled-collagen or synthetic peptide substrate, FALGPA. Assays were carried out in 50 mM Tris-HCl, 0.2 M NaCl, 5 mM CaCl_2_, pH 7.5 at 30 °C for FITC labeled-collagen, or 50 mM Tricine, 0.4 M NaCl, 40 mM CaCl_2_, pH 7.5 at 30 °C for FALGPA. Each collagenase was used at the amount of 0.5 μg for FITC labeled-collagen. When used for FALGPA, the amount of *G. hollisae* and *C. histolyticum* collagenase were 1.0 and 2.5 μg, respectively. The data represent the means three separate experiments. *P < 0.01; **P < 0.05, determined by unpaired t-test.

### Collagen cleavage assays comparing recombinant 62-kDa collagenase from *G. hollisae* with purified collagenase product from *C. histolyticum*

**Figure 3:**
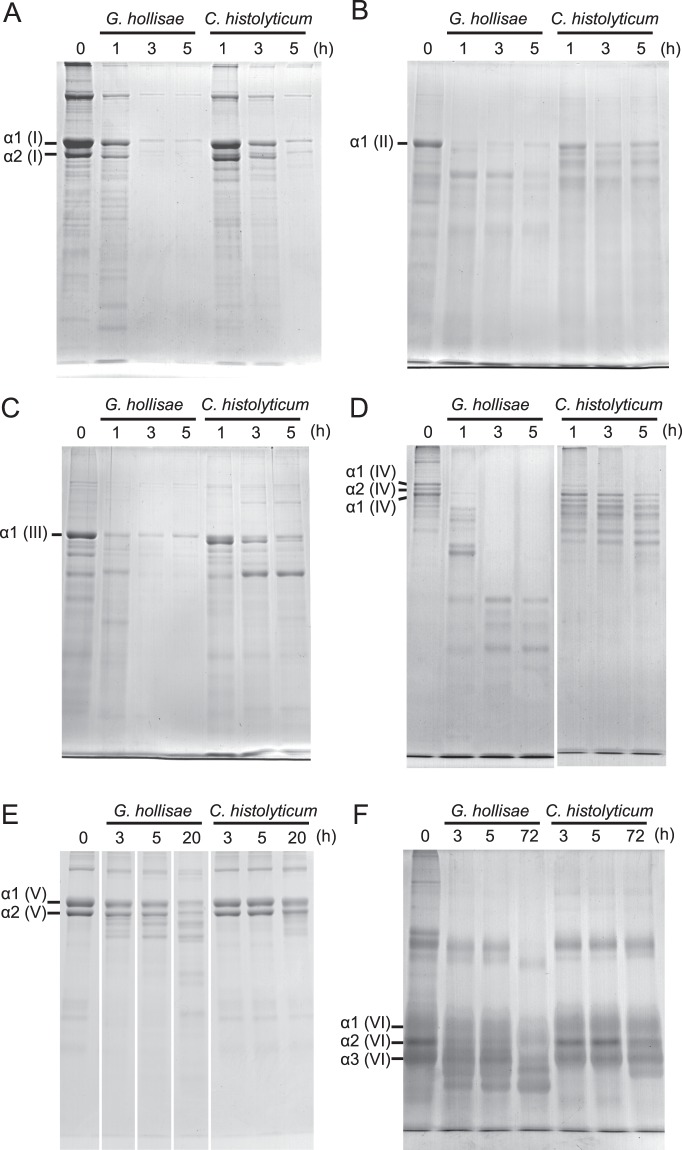
Collagen cleavage assay. Purified recombinant 62-kDa collagenase from *Grimontia hollisae* was incubated with type I (**A**), type II (**B**), type III (**C**), type IV (**D**), type V (**E**), and type VI (**F**) collagens at 30 °C. After the reaction was stopped by the addition of 1/4 volume of sodium dodecyl sulfate polyacrylamide gel electrophoresis (SDS-PAGE) sample buffer at each time point, each sample was analysed by SDS-PAGE using a 7.5% polyacrylamide gel. The uncropped gel is included in a Supplementary Information File.

To evaluate the substrate specificity of the recombinant 62-kDa collagenase from *G. hollisae*, we performed collagen cleavage assays using types I, II, III, IV, V, and VI collagens. The recombinant 62-kDa collagenase from *G. hollisae* cleaved all these types of collagens; it fully cleaved the alpha chains of types I, II, III, and IV collagens within 3 hours (Fig. 3A–D), type V collagen within 20 hours (Fig. 3E), and type VI collagen within 72 hours (Fig. 3F). In contrast, the purified collagenase product from *C. histolyticum* varied in cleavage activity based on collagen type; although it cleaved the alpha chains of types I, II, III, and IV collagens, it did not fully cleave these collagens within 5 hours (Fig. 3A–D), and it had not achieved considerable cleavage of type V collagen within 20 hours (Fig. 3E) or type VI collagen within 72 hours (Fig. 3F). Moreover, regarding types I, II, III, and IV collagens, the fragments of collagen cleaved by the recombinant *G. hollisae* collagenase appeared to be smaller than those cleaved by the purified collagenase product from *C. histolyticum* (Fig. 3A–D). Based on these results, we concluded that the recombinant *G. hollisae* collagenase exhibits higher cleavage activity for all these six types of collagens, compared with the purified collagenase product from *C. histolyticum*; however, it was unclear whether this difference was solely based on quantitative aspects of the enzyme activity, or on both quantitative and qualitative aspects.

### Evaluation of the suitability of recombinant 62-kDa collagenase from *G. hollisae* for use as a tissue dissociation enzyme by comparison with purified collagenase product from *C. histolyticum*

**Figure 4:**
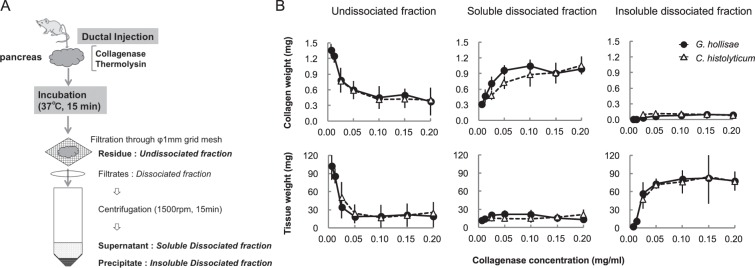
Quantification of collagen and tissue weights in the three fractions after pancreas dissociation. (**A**) Graphical representation of experimental procedure. (**B**) Undissociated, soluble dissociated, and insoluble dissociated fractions were prepared after pancreas dissociation using recombinant *Grimontia hollisae* collagenase (solid line) or purified *Clostridium histolyticum* collagenase (dashed line) with thermolysin (0.012 mg/ml). All fractions from collagenase-treated pancreata were hydrolysed by heating at 110 °C with 6 M HCl for 20 hours. Quantification of hydroxyproline content was performed using a 3200 QTRAP mass spectrometer; the collagen weight in each fraction was converted from hydroxyproline values. The total amino acid content of each fraction was measured with a L-8800 amino acid analyser; the tissue weight of each fraction was converted from the total amino acid content.

To evaluate the suitability of the recombinant 62-kDa collagenase from *G. hollisae* as a tissue dissociation enzyme, we used 21 whole murine pancreata as the dissociation target. We injected the recombinant *G. hollisae* collagenase into pancreatic ducts at concentrations of 0.00625, 0.0125, 0.025, 0.05, 0.10, 0.15, and 0.20 mg/ml, in combination with neutral protease thermolysin at a constant concentration of 0.012 mg/ml, to distend three pancreata for each collagenase concentration. We incubated all pancreata for 15 minutes at 37 °C, then divided the individual pancreata into three fractions: undissociated, soluble dissociated, and insoluble dissociated (Fig. 4A). The collagen weight in each fraction was estimated based upon the hydroxyproline amount determined by mass spectrometry; the tissue weight in each fraction was estimated based upon the total amino acid amount determined by amino acid analysis.

In undissociated fractions, as the collagenase concentration increased, the collagen weight and tissue weight decreased, such that they reached respective nadirs of 0.60 ± 0.12 mg and 12.29 ± 7.35 mg at the collagenase concentration of 0.05 mg/ml (Fig. 4B). In soluble dissociated fractions, as the collagenase concentration increased, the collagen weight increased; it reached a plateau of 0.96 ± 0.10 mg at the collagenase concentration of 0.05 mg/ml. In contrast, the tissue weight of soluble dissociated fractions remained low, near 14.18 mg. In insoluble dissociated fractions, as the collagenase concentration increased, the collagen weight remained low, near 0.06 mg; in contrast, the tissue weight increased, such that it reached a plateau of 48.00 ± 5.43 mg at the collagenase concentration of 0.05 mg/ml. Moreover, we found that the tissue dissociation tendencies of the purified collagenase product from *C. histolyticum* were comparable with those of the recombinant 62-kDa collagenase from *G. hollisae* (Fig. 4B). Based on these results, we concluded that the ability of the recombinant 62-kDa collagenase from *G. hollisae* to dissociate murine pancreata through digesting collagens within the pancreata is similar to that of the purified collagenase product from *C. histolyticum*; thus, we concluded that the 62-kDa recombinant *G. hollisae* collagenase could serve as a tissue dissociation enzyme.

### Evaluation of the suitability of recombinant 62-kDa collagenase from *G. hollisae* for use in isolation of functional islets from murine pancreata

**Figure 5:**
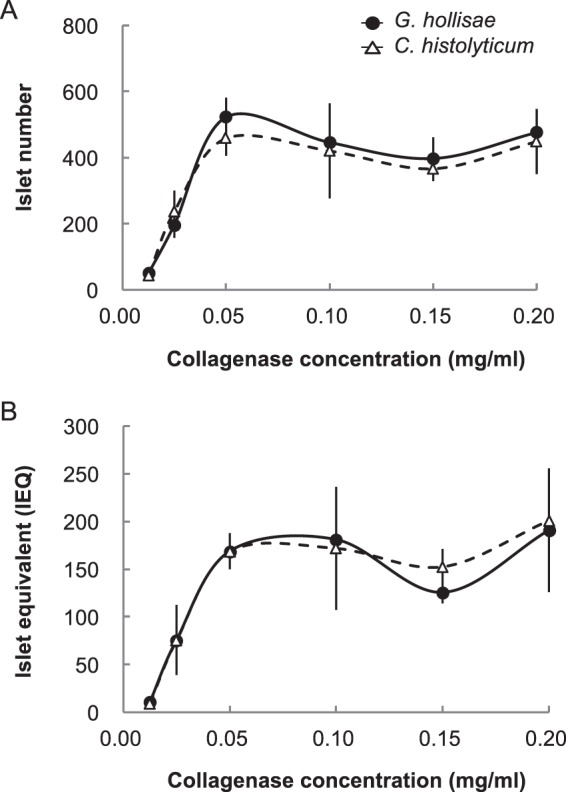
Determination of isolated islet number and islet equivalent. Pancreas dissociation was performed using recombinant *Grimontia hollisae* collagenase (solid line) or purified *Clostridium histolyticum* collagenase (dashed line) with thermolysin (0.012 mg/ml). Islets were purified from insoluble dissociated fractions using density-gradient centrifugation and stained with dithizone. Islet number (**A**) and islet equivalent (**B**) were determined based upon photos of all isolated islets in each fraction, which were taken using a charge-coupled device digital camera.

**Figure 6:**
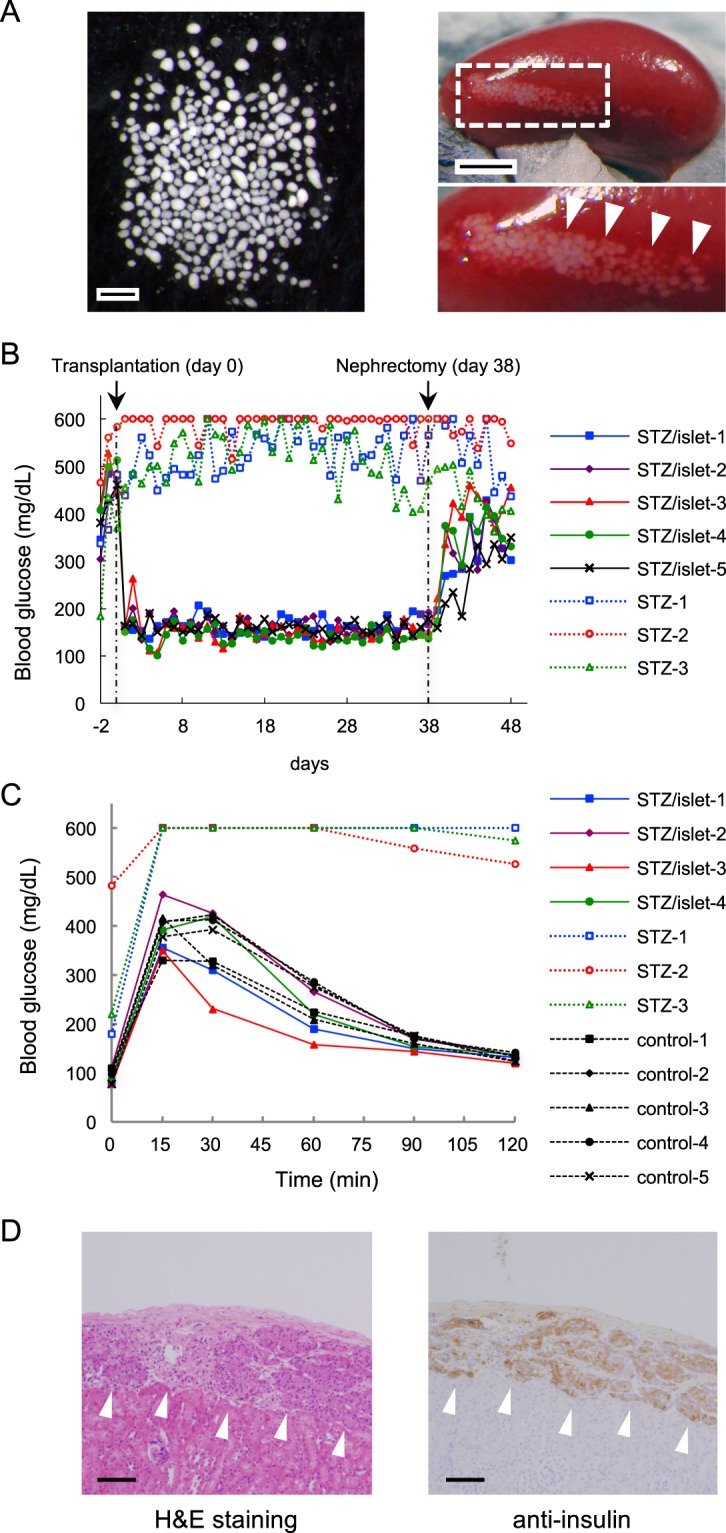
Transplantation of primary islet cells into diabetic mice. (**A**) Optical image of primary islet cells (left panel) and the transplantation of 300 islet cells into the subrenal capsular space of a recipient mouse (right panel). Scale bars, 500 μm (left); 2 mm (right). (**B**) Change in blood glucose concentrations of five diabetic mice that received 300 islet cells (solid lines) and three diabetic mice that did not undergo transplantation (dashed lines). Nephrectomy of the graft-bearing kidney was performed at 38 days after transplantation. (**C**) Intraperitoneal glucose tolerance tests of four diabetic mice that received 300 islet cells (solid lines), three diabetic mice that did not undergo transplantation (coloured dashed lines), and five healthy mice (black dashed lines) at 34 days after transplantation. (**D**) Histopathological analysis of sections of resected left kidney bearing transplanted islets in the subrenal capsule. Haematoxylin and eosin (H&E) staining (left panel) and immunohistochemical anti-insulin staining were performed. Scale bar, 100 μm.

To evaluate the suitability of the recombinant 62-kDa collagenase from *G. hollisae* for use in isolation of functional islets from murine pancreata, we used 18 whole murine pancreata as the isolation target. We injected the recombinant *G. hollisae* collagenase into pancreatic ducts at concentrations of 0.0125, 0.025, 0.05, 0.10, 0.15, and 0.20 mg/ml, in combination with neutral protease thermolysin at a constant concentration of 0.012 mg/ml, to distend three pancreata for each collagenase concentration. We incubated all pancreata for 15 minutes at 37 °C to dissociate them, then purified the dissociated tissue by using discontinuous density gradients. The purified islets were stained with dithizone and served to evaluate islet number and islet equivalent. As the concentration of collagenase increased, both islet number and islet equivalent increased, reaching respective plateaus of approximately 500 islets and 170 islet equivalent at the collagenase concentration of 0.05 mg/ml (Fig. 5A,B). Moreover, we found that the islet isolation tendencies of the purified collagenase product from *C. histolyticum* were comparable with those of the recombinant 62-kDa collagenase from *G. hollisae* (Fig. 5A,B). Thereafter, to confirm the function of the islets isolated using the recombinant 62-kDa collagenase from *G. hollisae*, we performed a transplant bioassay in five mouse recipients; this assay is recognised as the gold standard for islet function evaluation^26^. Isogeneic islets were isolated using the recombinant 62-kDa collagenase from *G. hollisae* and thermolysin at concentrations of 0.15 mg/ml and 0.012 mg/ml, respectively. Three hundred purified islets were transplanted under the kidney capsule of each mouse recipient; diabetes had been induced in all mice by prior administration of streptozotocin (Fig. 6A). The blood glucose concentrations gradually decreased in all mice that received islets, such that they returned to normal within 3 days after transplantation; conversely, diabetic mice that did not receive islets continued to exhibit high concentrations of blood glucose. All mice that had achieved normoglycaemia reverted to hyperglycaemia immediately after the removal of kidneys bearing islet grafts, at 38 days after transplantation (Fig. 6B). In four of the five mice that received islet grafts 34 days after transplantation, intraperitoneal glucose tolerance tests showed that the mice demonstrated nearly normal responses (Fig. 6C). Histological analysis using immunohistochemical staining for insulin revealed that insulin-positive islet grafts with well-preserved islet structure were present under the kidney capsules (Fig. 6D). Based on these results, we concluded that the ability of the recombinant 62-kDa collagenase from *G. hollisae* to isolate functional murine islets is similar to that of the purified collagenase product from *C. histolyticum*^28^.

## Discussion

In this study, we have shown that the 62-kDa collagenase protein from *G. hollisae* is successfully generated using a recombinant technique so as to meet the conditions to be used as an enzyme product for research and therapeutic applications, and that it possesses sufficient collagenase activity for isolation of primary cells. The evidence showing that the conditions of the generated 62-kDa recombinant protein from *G. hollisae* are met to be used as an enzyme product is that the recombinant protein exists in monomeric form (Fig. 2A); furthermore, it does not exhibit protein aggregation (Fig. 2B) when expressed both in stable transformants and in the *Brevibacillus* Expression System on the litre scale, and subsequently purified using ion-exchange chromatography. In terms of the collagenase activity of the recombinant protein being sufficient to isolate primary cells, the evidence is that the recombinant protein not only dissociates murine whole pancreata but also isolates functional islets with collagenase activity similar to that of a purified collagenase product from *C. histolyticum* when used with neutral protease thermolysin (Figs. 4, 5 and 6).

In addition to sufficient collagenase activity, we found that the 62-kDa recombinant protein from *G. hollisae* possessed stability in collagenolytic activity (Fig. 2E). There are two factors that could explain this stability. First, the recombinant 62-kDa collagenase from *G. hollisae* does not undergo attack by other proteases that would exist as impurities, because our production process yields a highly purified protein (Fig. 2A,B). Second, the recombinant 62-kDa collagenase from *G. hollisae* also does not undergo auto-digestion; this is potentially because it lacks non-specific protease activity (it exhibits no caseinase activity or cytotoxicity [Fig. S2]), and/or because it has low sensitivity to collagenase attack (all collagenous sequences of G-X-Y repeats in the recombinant 62-kDa collagenase from *G. hollisae* were less subject to collagenase attack than the cleavage site in the recombinant 74-kDa collagenase from *G. hollisae* [Fig. S3C]). Notably, the 62-kDa recombinant collagenase from *G. hollisae* contains four collagenous sequences: three in the linker region and one in the catalytic domain (Figs. S3A,B). Based on our quadrupole time-of-flight mass spectrometry findings (Fig. S1), the collagenous sequence in the catalytic domain is less sensitive than the sequences in the linker region. To clarify the mechanism underlying this difference in sensitivity, we performed homology modelling of the recombinant 62-kDa collagenase from *G. hollisae* using the crystal structure of clostridial collagenase ColG as a template^29^; we found that the collagenous sequence in the catalytic domain might be located on an inner surface that is not exposed to interactions with the external environment (Fig. S4B). The environment around the collagenous sequence in the catalytic domain might contribute to avoidance of collagenase attack.

The stable collagenolytic activity of recombinant *G. hollisae* collagenase has been achieved by removing the PPC domain from the 74-kDa recombinant protein and producing the remaining 62-kDa recombinant protein (Figs. 1 and 2). The instability of the 74-kDa recombinant protein (Fig. 1D) should be attributed to the protein structure, where the catalytic domain and PPC domain are connected by a linker containing a collagenous sequence that is highly sensitive to auto-degradation (Figs. 1A and S3C). The collagenolytic activity of the 62-kDa recombinant protein is provided by its single catalytic domain. Several proteins that consist solely of a catalytic domain are reported to function as collagenases^29–32^. To the best of our knowledge, the 62-kDa recombinant *G. hollisae* protein is currently the only collagenase that consists solely of a catalytic domain and can dissociate murine whole organs for isolation of primary cells. The 74-kDa recombinant protein possesses greater collagenolytic activity than the 62-kDa recombinant protein (Fig. 1C,E), presumably because the PPC domain contains a collagen-binding site that enhances collagenolysis^24^. Matsushita *et al*. reported that clostridial collagenase ColH, which lacks a collagen-binding domain and consists solely of a catalytic domain, exhibits collagenolytic activity; however, this activity is reduced to 0.05-fold that of intact ColH with the collagen-binding domain^31^. Because our current system could not produce a recombinant *G. hollisae* collagenase preparation that solely comprises the 74-kDa recombinant protein, a direct comparison of collagenolytic activity between 74-kDa and 62-kDa recombinant proteins could not be performed. However, our findings regarding the comparison of collagenolytic activity between the 74-kDa recombinant protein—with a small amount of spontaneously truncated 62-kDa protein—and the 62-kDa recombinant protein suggest that the collagenolytic activity of the 74-kDa recombinant protein is approximately two-fold greater than that of the 62-kDa recombinant protein (Fig. 1C). Moreover, the collagenolytic activity was not proportional to the ratio of recombinant proteins in the 74-kDa recombinant protein preparation (Fig. 1E). This lack of direct proportionality is potentially because the 74-kDa and 62-kDa recombinant proteins might not function independently to digest collagen; the 62-kDa recombinant protein might possess greater cleavage activity for denatured collagen than for intact collagen. Thus, when the 74-kDa recombinant protein cleaves and denatures collagen, the 62-kDa recombinant protein reacts more readily with the denatured collagen, rather than the remaining intact collagen. This hypothesis is supported by our findings that the specific activity of the 62-kDa recombinant protein was higher for denatured collagen than for intact collagen (Fig. S5).

To evaluate whether the 62-kDa recombinant collagenase can serve as a tissue dissociation enzyme, we used murine pancreata as the target organ. For this evaluation, we developed a method that could directly assess both the ability of the collagenase to dissociate the murine pancreata and its collagen-digesting activity in the pancreata. This method can determine the tissue weight and collagen weight in the undissociated, soluble dissociated, and insoluble dissociated fractions after enzyme treatment (Fig. 4A). Tissue weight in the insoluble dissociated fraction, relative to tissue weight in the undissociated fraction, represents the activity of collagenase as a tissue dissociation enzyme. Collagen weight in the soluble dissociated fraction, relative to collagen weight in the insoluble dissociated fraction, represents the direct contribution of collagenase made to tissue dissociation, as collagen becomes soluble after it is cleaved^33^. Typically, the activity of collagenase to dissociate pancreas tissue has been evaluated based upon the amount of isolated primary islets^27,34^. However, this evaluation system might not be suitable for assessment of the direct contribution of collagenase to pancreas dissociation. The amount of isolated primary islets could be influenced by factors other than the collagenase, such as the conditions of the islet isolation procedure, including the pressure during collagenase injection through the duct to distend the pancreas^35^, as well as the technique for islet purification^36,37^. In this study, collagenase activity was evaluated using tissue weight and collagen weight in each of the three fractions after collagenase treatment (Fig. 4B); the findings were consistent with the collagenase activity determined based upon the amount of isolated primary islets (Fig. 5). These results indicate consistency in our islet isolation technique. The evaluation method we have developed here could serve as a useful *ex vivo* assay system to determine whether collagenase products are suitable for tissue dissociation.

When considering clinical uses for the recombinant 62-kDa collagenase from *G. hollisae*, several advantages may exist, compared with conventional purified clostridial collagenase products. The recombinant *G. hollisae* collagenase is able to digest type VI collagen, whereas the purified clostridial collagenase is not (Fig. 3F); these findings are consistent with the results of previous studies^38,39^. Because type VI collagen is ubiquitous in animal tissues^40^ and may increase during fibrous changes (e.g., liver fibrosis)^41^, the ability to digest type VI collagen could enable effective isolation of primary cells from fibrous tissues. Because pancreatic tissue contains type VI collagen^42^, the recombinant *G. hollisae* collagenase might provide better yields of isolated islets from a fibrous pancreas, compared with conventional purified clostridial collagenase products; this could improve the status of islet autotransplantation in terms of controlling blood glucose concentrations^43^.

In conclusion, we have successfully produced a stable recombinant collagenase from *G. hollisae* as a 62-kDa protein through direct expression with the Brevibacillus Expression System. We provide evidence that this recombinant *G. hollisae* collagenase cleaves representative types of collagen in tissues, and that it dissociates murine pancreata for isolation of functional islets through digestion of pancreas collagens. These findings suggest that this recombinant *G. hollisae* collagenase may be a useful tool for dissociation of tissue to isolate primary cells in clinical settings.

## Methods

### Reagents

Liberase MTF C/T, GMP grade kit (Roche, Basel, Switzerland) was used as the purified clostridial collagenase and thermolysin product. Bovine pepsin-solubilised types I, II, III, and V collagens (Nippi Inc., Tokyo, Japan), bovine acid soluble type IV collagen (Nippi Inc.), and human pepsin-solubilised type VI collagen (BD Biosciences, Bedford, MA, USA) were used as substrates for collagen cleavage assays. *Brevibacillus* expression vector pNY326 and *Brevibacillus choshinensis* strain HPD31-SP3 (Takara Bio, Shiga, Japan) were used for expression of recombinant proteins.

### Preparation of recombinant proteins from *G. hollisae*

The nucleotide sequence of *G. hollisae* collagenase was obtained from the DDBJ database (AB600550). The recombinant 74-kDa collagenase was expressed and purified as previously described^23^. To express C-terminal truncated collagenase, two types of truncated collagenase gene fragments were amplified, Col62k (62 kDa, 1677 bp) and Col60k (60 kDa, 1611 bp), using the following primers: Col62k forward, 5′-CCCATGGCTTTCGCTGCGGTTGAACAGTGTGATCT-3′; Col62k reverse, 5′-CATCCTGTTAAGCTTAGGTATTACCACCAGATTCA-3′, Col60k forward, 5′-CCCATGGCTTTCGCTGCGGTTGAACAGTGTGATCT-3′; Col60k reverse, 5′-CATCCTGTTAAGCTTACTGTCGCCCTTCGCCAGC-3′. To amplify linearised vector pNY326 DNA sequence, the following primers were used: forward, 5′-AAGCTTAACAGGATGCGGGG-3′; reverse, 5′-AGCGAAAGCCATGGGAGCAA-3′. The underlined sequences indicate overlap with both ends of insert fragment sequences. All PCR reactions were conducted using the Expand High Fidelity PCR System (Roche). Insert fragments amplified by PCR were mixed with the linearised vector pNY326; this mixture was transformed into competent cells to construct recombinant expression plasmids using the *Brevibacillus in vivo* cloning method. *Brevibacillus* transformants were cultured, and the collected supernatants were then purified as previously described^23^.

### SDS-PAGE

SDS-PAGE was performed using a 7.5% or 10% polyacrylamide gel, in accordance with the Laemmli method^44^.

### Assay for collagenolytic activity

The collagenolytic activity of collagenase was measured using FITC-labelled type I collagen, as previously described^45^. One unit of collagenolytic activity was defined as the amount that degraded 1 μg of FITC-labelled collagen at 30 °C per minute. Protein concentrations were determined using Coomassie Plus – The Better Bradford^TM^ Assay Reagent (Thermo Fisher Scientific, Rockford, IL, USA).

### Stability analysis of collagenase

Collagenase (0.5 mg/ml) was incubated at 37 °C in 50 mM Bis-Tris-HCl buffer (pH 7.5) containing 0.2 M NaCl and 5 mM CaCl_2_. After incubation for various time intervals, the reaction mixture was analysed by SDS-PAGE and its collagenolytic activity was measured using FITC-labelled type I collagen, as described above.

### Real-time gelatin zymography

Real-time gelatin zymography was performed as previously described^46^. Collagenase was subjected to SDS-PAGE using a 10% gel containing 0.05% FITC-labelled gelatin under non-reducing conditions.

### Size exclusion chromatography

Size exclusion chromatography of collagenase was performed on an Alliance 2895 system (Waters, Milford, MA, USA), using a Superdex 200 HR10/30 column (GE Healthcare). Samples were loaded onto the column and eluted in an isocratic manner with 50 mM Bis-Tris-HCl (pH 7.5) containing 0.2 M NaCl, at a flow rate of 0.75 ml/minute. Separated protein fractions were detected at 220 nm.

### Determination of pH- and temperature-dependence of collagenase

The influence of pH on collagenase activity was determined as follows. Collagenase and a specific collagenase substrate, FALGPA (Bachem AG, Bubendorf, Switzerland), were preincubated at 30 °C for 10 minutes in various buffers. The following buffers were used: 50 mM MES (pH 6.0–7.0), 50 mM HEPES (pH 7.0–8.5), 50 mM TAPS (pH 8.5 and 9.0), and 50 mM CHES (pH 9.0 and 10.0) containing 0.2 M NaCl and 5 mM CaCl_2_. The FALGPA assay was performed in accordance with a modified version of a previously reported method^23,47^. One unit of activity was defined as the amount that degraded 1 μmol of FALGPA peptide at 30 °C per minute.

The temperature-dependence of collagenase activity was measured by using the Wünsch method^48^. A specific collagenase substrate, Pz-Pro-Leu-Gly-Pro-D-Arg-OH (Bachem AG, Bubendorf, Switzerland), was used. Collagenase (0.13 μg/ml) was incubated with the Pz peptide in 50 mM HEPES (pH 7.5) containing 0.2 M NaCl and 5 mM CaCl_2_ at various temperatures (10–60 °C). Aliquots of the reaction mixture at 2 minutes, 4 minutes, 8 minutes, and 12 minutes were mixed with equal volumes of 25 mM citrate to stop the reaction. After extraction into ethyl acetate, the amount of liberated Pz-Pro-Leu was determined by absorbance at 320 nm, measured using a spectrophotometer, then used to calculate the collagenase activity per minute.

### Kinetic analysis

Kinetic analysis was performed using FITC-labelled type I collagen and FALGPA, as previously described^23^. Briefly, 0.5 μg of collagenase was incubated with various amounts of FITC-labelled type I collagen (10–50 μg) at 30 °C for 5 minutes. FALGPA concentrations ranged from 0.5 mM to 3.0 mM. V_max_ and K_m_ values for hydrolysis of native collagen and FALGPA were estimated with a Lineweaver–Burk plot, using the reaction rates at different substrate concentrations.

### Collagen cleavage assay

Bovine type I, II, III, IV, and V collagens (each 1 mg/ml in 5 mM acetic acid) were mixed with an equal volume of neutralising buffer solution (0.1 M Tris-HCl [pH 7.5] containing 0.4 M NaCl and 10 mM CaCl_2_). Human type VI collagen was dissolved in 50 mM Tris-HCl (pH 7.5) containing 0.2 M NaCl and 5 mM CaCl_2_ to prepare 0.5 mg/ml solution. Then, 1/10 volume of collagenase (10 μg/ml) was added to each collagen solution and incubated at 30 °C for the time intervals shown in Fig. 3. To stop the reaction, 1/4 volume of SDS sample buffer was added. Each sample was then cooled on ice immediately and analysed by SDS-PAGE using a 7.5% polyacrylamide gel.

### Animals

Murine pancreatic islets were obtained from 8-week-old male inbred C57 BL/6 NCrSlc mice (Sankyo Laboratory, Tokyo, Japan) weighing 20 to 27 g. All animals used in this study were handled in accordance with the Guide for the Care and Use of Laboratory Animals published by University of Tokyo; the animal study protocol was approved by the committee for animal experiments and related activities at University of Tokyo (approved protocol ID: 25–6). All surgeries were performed under anaesthesia and all possible efforts were made to minimise suffering.

### Quantification of collagen weight and tissue weight in the three fractions after pancreas dissociation

The undissociated, soluble dissociated, and insoluble dissociated fractions were prepared after pancreas dissociation using collagenase and thermolysin; the undissociated fraction was the remnant on a φ1-mm grid mesh, the soluble dissociated fraction was the supernatant after centrifugation (490 × g, 20 minutes) of the fraction that had passed through the mesh, and the insoluble dissociated fraction was the precipitate that remained after the above centrifugation (Fig. 4A). All fractions from collagenase-treated pancreata were hydrolysed by heating at 110 °C with 6 M HCl for 20 hours. The collagen content in each fraction was evaluated based on the amount of hydroxyproline, which is a specific amino acid in collagen. Quantification of hydroxyproline content was performed on a hybrid triple quadrupole/linear ion trap 3200 QTRAP mass spectrometer (AB Sciex, Foster City, CA, USA) coupled to an Agilent 1200 Series HPLC system (Agilent Technologies, Inc., Santa Clara, CA, USA). To correct the ionisation efficiency changes among different types of samples, stable isotope-labelled collagen was used^49^. Acid hydrolysate of stable isotope-labelled collagen containing ^13^C_5_^15^N_1_-hydroxyproline was added to the hydrolysed pancreatic samples and hydroxyproline standards (Wako Chemicals, Osaka, Japan) as an internal standard, prior to mass spectrometric analysis^50^. Separately, the hydroxyproline weight and total amino acid content in murine skin type I collagen were calculated based on the amino acid composition of the collagen (Table S1). Based upon the calculation that hydroxyproline constituted 12.6% of the collagen weight in murine type I collagen, the collagen weight in each fraction was converted from hydroxyproline values by using the following coefficient: 7.94 (i.e., 100%/12.6%). The tissue weight was estimated by the total amount of amino acids. The total amino acid content of each fraction was measured with a L-8800 amino acid analyser (Hitachi, Tokyo Japan). The dry weight of murine pancreas was measured after fat removal by hexane treatment (Table S2). Based upon the calculation that total amino acids constitute 61.1% of the dry weight of murine pancreas after fat removal, the tissue weight of each fraction was converted from the total amino acid content by using the following coefficient: 1.64 (i.e., 100%/61.1%).

### Determination of isolated islets number and islet equivalent

Islets were purified from insoluble dissociated fractions using density-gradient centrifugation with ET-Kyoto solution (Otsuka Pharmaceutical, Tokyo, Japan) and OptiPrep (Axis-Shield PoC, Oslo, Norway), then stained with dithizone (diphenylthiocarbazone, Wako Pure Chemical Industries, Osaka, Japan). Islet number and islet equivalent were determined based upon photos of all isolated islets in each fraction, which were taken using a charge-coupled device digital camera (Olympus DP71, Olympus, Tokyo, Japan) attached to an inverted microscope (Olympus IX81), using a tiling software (Meta Morph, Molecular Device, San Jose, CA, USA). To determine islet volume, assuming that each islet is a sphere, islets were manually extracted using Photoshop (Adobe, San Jose, CA, USA); the projection area A of each islet was measured using ImageJ software (NIH, Bethesda, MD, USA) and the islet equivalent was estimated by determining islet volume V (i.e., V = [4/3√π] A^1.5^). Islet equivalent was regarded as the standard estimate of isolated islet volume. One islet equivalent was determined to correspond to the tissue volume of a perfectly spherical islet with a diameter of 150 μm^28,51^.

### Islet transplantation

Three hundred islets were transplanted into the renal subcapsular space of streptozotocin-induced diabetic mice (n = 5), using a previously described method^52^. In these mice, diabetes was induced by intraperitoneal administration of streptozotocin (Sigma-Aldrich Co., St. Louis, MO, USA) (120 mg/kg). Blood glucose levels were measured every day; mice with non-fasting blood glucose levels >400 mg/dl in two consecutive measurements were considered diabetic. After transplantation, mice with non-fasting blood glucose levels <200 mg/dl in two consecutive measurements were regarded as normoglycaemic, which was indicative of successful transplantation of a functioning graft. Nephrectomy of the graft-bearing kidney was performed 38 days after transplantation to confirm that islet grafts directly contributed to the normalisation of blood glucose after transplantation.

### Intraperitoneal glucose tolerance tests

Intraperitoneal glucose tolerance tests were performed 34 days after islet transplantation. After a 14-hour fast, D-glucose (2.0 g/kg) was infused intraperitoneally as a single bolus; blood glucose concentrations were determined before glucose injection, as well as at 15, 30, 60, 90, and 120 minutes after injection. The results of intraperitoneal glucose tolerance tests were evaluated by the area under the curve and Kg values. Five naive C57BL/6 male mice were tested as controls for this assay.

### Histological analysis

Removed islet-bearing kidneys were fixed in 4% paraformaldehyde and embedded with paraffin. The sections were stained with haematoxylin/eosin. For immunohistochemical analysis, guinea pig anti-insulin antibody (Dako, Glostrup, Denmark) and horseradish peroxidase-labelled anti-guinea pig antibody (Dako) were used to detect insulin as primary and secondary antibody, respectively.

### Statistical analysis

All data are presented as mean ± standard deviation. Statistical analyses were performed by using unpaired t-tests (Microsoft Excel, Microsoft, Redmond, WA, USA) for comparisons between two groups and by using one-way ANOVA (ANOVA4 on the Web, https://www.hju.ac.jp/kiriki/anova4/index_js.html) for comparisons among four groups. A value of p < 0.05 was considered statistically significant.

## Supplementary information

Supplementary information_TANAKA.

## Data Availability

The data are available from the corresponding author on reasonable request.
