# Supplementary material for: Recombinant collagenase from Grimontia hollisae as a tissue dissociation enzyme for isolating primary cells
Source: Sci Rep. 2020 Mar 3;10:3927. doi: 10.1038/s41598-020-60802-z (PMC7054364; doi:10.1038/s41598-020-60802-z)
Supplement: Supplementary file 1 — Supplementary information_TANAKA. [file 41598_2020_60802_MOESM1_ESM.pdf]

Supplementary Information

Recombinant collagenase from *Grimontia hollisae* as a tissue dissociation enzyme for isolating primary cells

Keisuke Tanaka[1*], Teru Okitsu[2*], Naoko Teramura[1], Katsumasa Iijima[1], Osamu Hayashida[1], Hiroki Teramae[3] and Shunji Hattori[1]

[1] Nippi Research Institute of Biomatrix, Toride, Ibaraki 302-0017 Japan
[2] Institute of Industrial Science, The University of Tokyo, Meguro, Tokyo 153-8904, Japan
[3] Faculty of Teacher Education, Shumei University, Yachiyo, Chiba 276-0003, Japan

This PDF file includes:

- Figs. S1 to S5
- Tables S1 to S2
- Supplementary information (full-length gels for Figs. 1C, 1E, 3D and 3E)

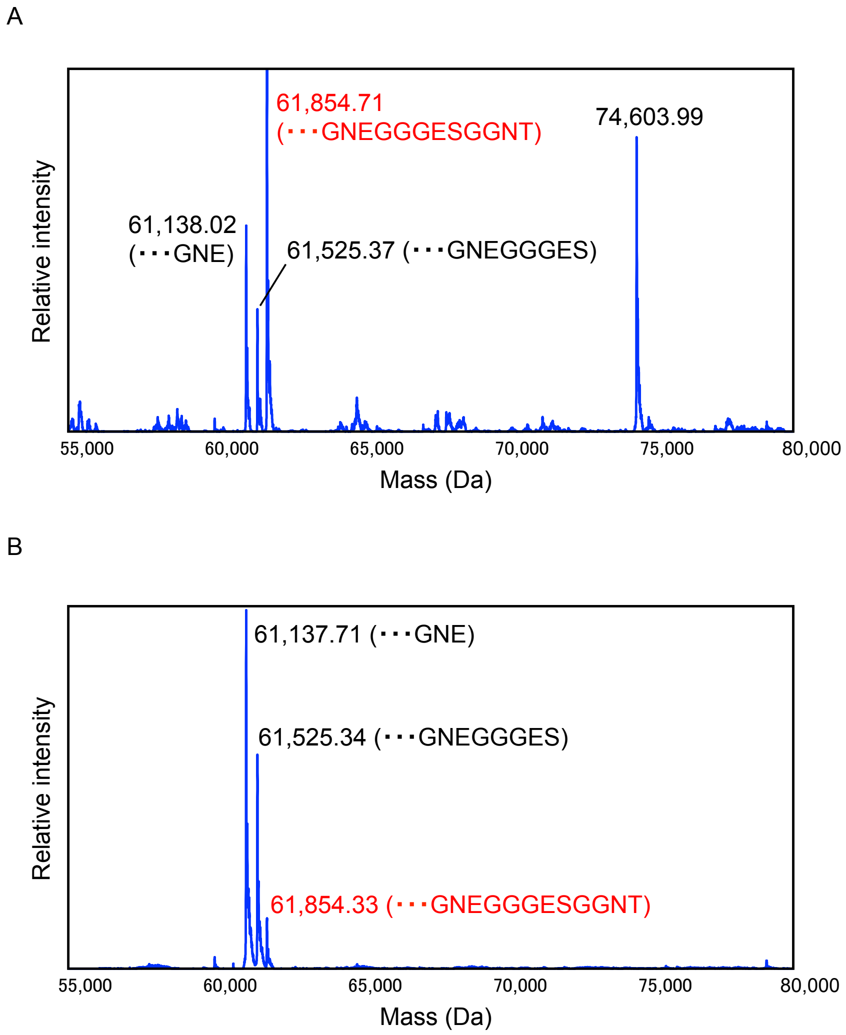

Fig. S1. Deconvoluted mass spectrum of recombinant 74-kDa collagenase containing spontaneously truncated ~60-kDa protein (A) and recombinant 62-kDa collagenase (B).

The collagenases desalted with ultrafiltration with a 3 kDa cut-off (Amicon Ultra) was diluted with 0.1% formic acid/50% methanol and subjected to direct infusion analysis using a QTOF mass spectrometer (maXis II). The obtained mass spectrum was deconvoluted using Compass DataAnalysis version 4.3 (Bruker Daltonics) with the Maximum Entropy algorithm. The molecular masses and C-terminal amino acid sequences are indicated.

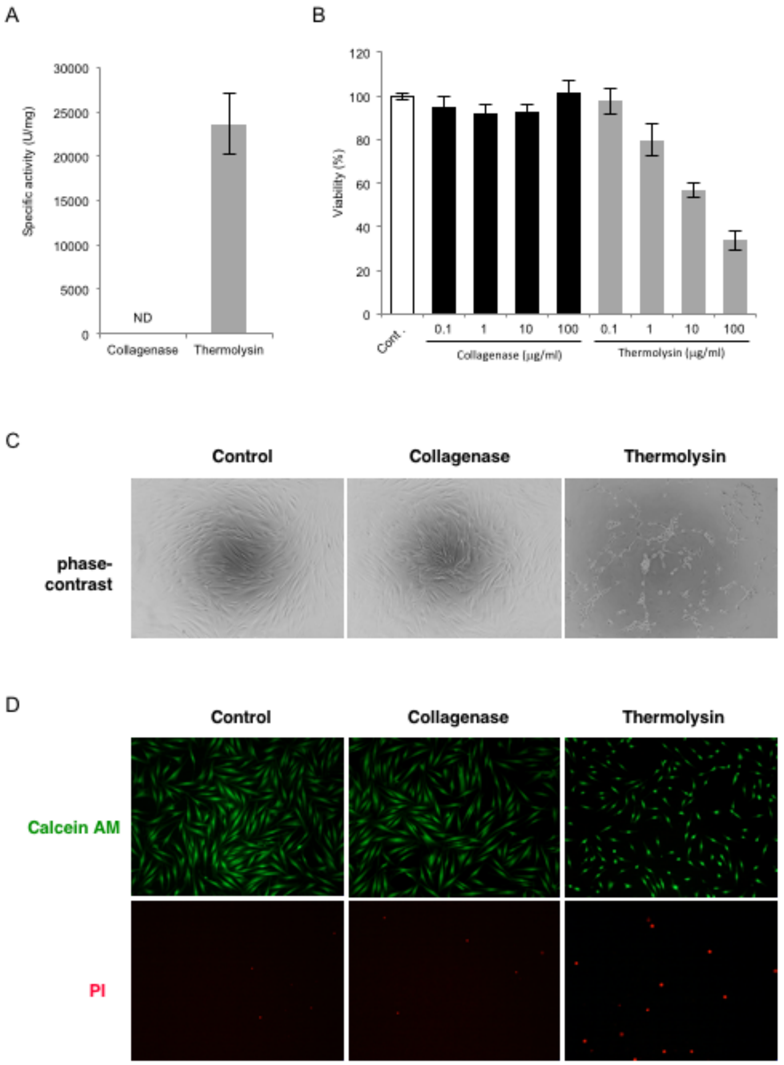

Fig. S2. Caseinase activity and cytotoxicity of the recombinant *G. hollisae* collagenase.

(A) The enzyme solution (collagenase or thermolysin) was mixed with 50 mM Tis-HCl/ 0.2 M NaCl/ 5 mM $CaCl_2$ (pH 7.5) containing 0.05% FITC-casein and incubated for 30 min at 30 ℃. After adding EDTA to stop the enzymatic reaction, the fluorescence intensity (530 nm Em/485 nm Ex) of the degraded FITC-labeled fragments was measured. The values represent the average of triplicate trials±SD.

(B) Cell viability of human foreskin fibroblasts treated with collagenase or thermolysin. Cells were seeded in 96 well plate and cultured in 10% FBS/ DMEM. When cells become confluent, the enzyme solution (collagenase or thermolysin) was added to each well and the plate was incubated for 15 hours. After incubation, MTT assay was performed according to the manufacture's instruction (Cell Counting Kit-8, Dojin Chemical). The values represent the average of triplicate trials±SD.

(C) The phase-contrast images of fibroblasts before MTT assay.

(D) Live-dead images of enzyme-treated fibroblasts. Cells cultured on a slide glass were incubated with 0.5 mM calcein AM (Dojin Chemical) and 2 mM propidium iodide (PI) (P-4170, Sigma-Aldrich) for 1 hour prior to imaging. Living cells and dead cells are stained by calcein-AM (green) and PI (red), respectively.

A

```
  1 MELKALALTVSALLVGQSVYASEVFAHPGMPVQQKLQPNLLQQSTRLQPEQHIHGLERTDRQYRPTDATQ  70
 71 QPEPPTLLKRQVSVQQDAVEQCDLSQFQTTSSNQLMAAIRQQGASCVNALFSADTGVQEAAFSSNHMYNV 140
141 AQYTRTLAQQYAGGGSDELEALYLYLRAGYYAEFYNSNITFLSWVTPAVKGAVDAFVQNAHFYDNGDAHG 210
211 KVLNEVIITMDSAGLQHAYLDVVTQWLTRWNAQYAEHWYMRNAVNGVFTLLFGGQWNNQYTSLIGEQTAL 280
281 VTALQAFALDRTKVNSPTEFMAANAARELGRLARYTDATIAPKVTEGLTAIFGQYPSYGDGDAIWLGAAD 350
351 TASYYADCSQFNICGFEDALRDAALNQTFICSDTIKIRSQDMSQAQHLAACDKMAYEESFFHTTLETGNQ 420
421 PVADDHNTQLQVNIFNSDTDYGKYAGPIFGIDTNNGGMYLEGNPANVGNIPNFIAYEASYANPDHFVWNL 490
491 EHEYVHYLDGRFNMYGDFGTPTELVVWWSEGVAEYVSRVNDNPQAIATIQDGSTYTLAQVFDTTYDGFDV 560
561 DRIYRWGYLAVRFMFERHPDEVQRMLSATRQGRWAEYKAIISGWANQYQSEFAQWTEALAKGDSGAGNGE 630
631 GTGSGNEGGGESGGNTGLPENCAVLPKISDGRLALDEAACLADTASASDVLWFSIPAVSEYQTIAITAGN 700
701 GTGDLTLEYSNLNWPDGTNVQASSANMGNSECIILEHQANYWGYLKVSGSFENAALLVEAGSNQCRQ    767
```

B

Amino acid	Sequence	Location in 74 kDa recombinant collagenase
506-511	GDFGTP	Catalytic domain
622-627	GDSGAG	Linker
635-640	GNEGGG	Linker
640-645	GESGGN	Linker
644-649	GNTGLP	Linker (Cleavage site)

C

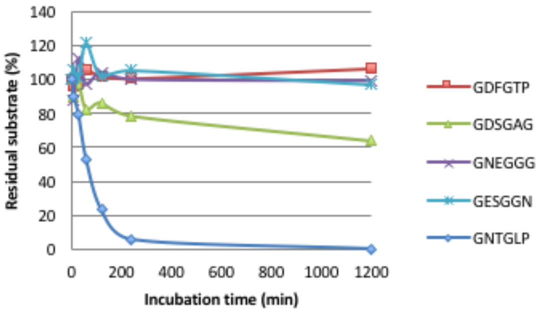

Fig. S3. Susceptibility of GXY repeats to the recombinant *G. hollisae* collagenase.

(A) Amino acid sequence of *G. hollisae* collagenase. Pre-pro region, catalytic domain and PPC domain are shown in gray, blue and red, respectively. The GXY repeats are underlined.

(B) Location of GXY repeats in 74-kDa recombinant collagenase.

(C) Each peptides of GXY repeats (10 mg/ml) was incubated in 50 mM Tris-HCl/ 10 mM $CaCl_2$ buffer (pH 7.5) with 500 ng of the recombinant collagenase at 37 ℃, followed by LC-MS analysis using 1200 (Agilent Technologies) and 3200QTRAP (SCIEX). The data are expressed as relative values, with the amount of substrate before incubation being 100 percent.

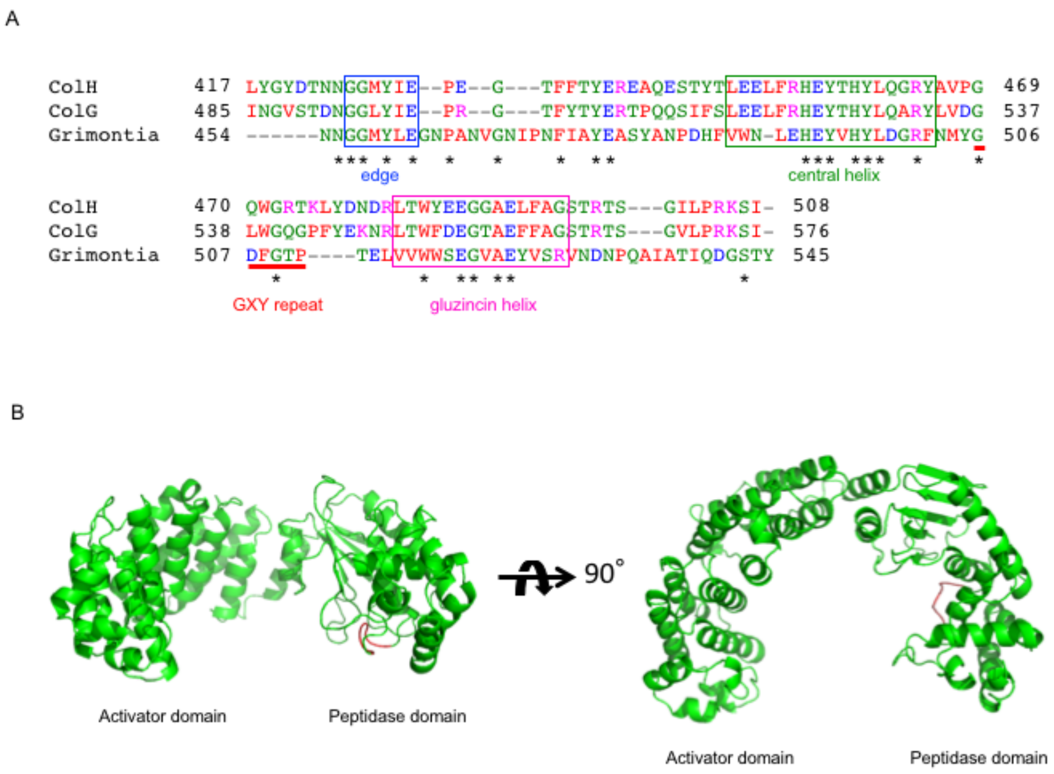

Fig. S4. Sequence alignment of *G. hollisae* collagenase with *C. histolyticum* collagenases and catalytic domain model of *G. hollisae* collagenase.

(A) The amino acid sequences close to catalytic center from *G. hollisae* (aa 454-545, NCBI accession number: BAK39964), *C. histolyticum* collagenase ColH (aa 417-508, NCBI accession number: AB014075), and *C. histolyticum* collagenase ColG (aa 485-576, NCBI accession number: D87215) were aligned by using the CLUSTAL Omega program. Identical residues among the three sequences are indicated by asterisks. Boxes indicate edge strand, central helix and glizincin helix in *C. histolyticum* collagenases shown in blue, green and magenta, respectively. GXY repeat is underlined in red.

(B) Homology model of the catalytic domain of *G. hollisae* collagenase was constructed with SWISS-MODEL server, based on crystal structure of the catalytic domain of *C. histolyticum* collagenase ColG (Protein Data Bank: 2Y3U). The GDFGTP sequence are highlighted in red.

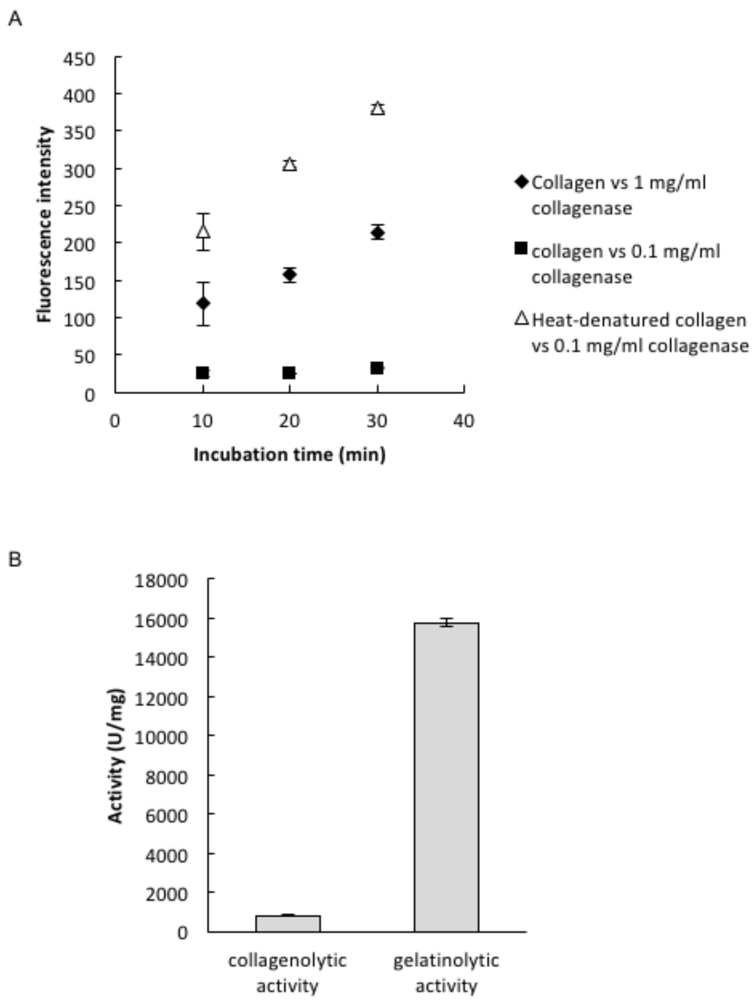

Fig. S5. Comparison between collagenolytic and gelatinolytic activities of the recombinant *G. hollisae* collagenase.

(A) The collagenase solution was mixed with 50 mM Tis-HCl/ 0.2 M NaCl/ 5 mM $CaCl_2$ (pH 7.5) containing 0.05% FITC-collagen or FITC-gelatin, and incubated at 30 ℃. After adding EDTA to stop the enzymatic reaction at various time points, the fluorescence intensity (530 nm Em/485 nm Ex) of the degraded FITC-labeled fragments was measured. The values represent the average of triplicate trials±SD.

(B) The collagenolytic and gelatinolytic activities of the recombinant collagenases were determined using FITC-collagen and FITC-gelatin, respectively. The values represent the average of triplicate trials±SD.

Table S1. Amino acid composition of murine type I collagen.
The weights of hydroxyproline and total amino acids in murine skin type I collagen were calculated from its amino acid composition to find out the correspondence of these values. Collagen weight and Hyp coefficient were calculated using numerical formulas as described below.
Collagen weight (g) = (Total amino acids content) – 999 x H_2O =108927.5 – 999 x 18 = 90945.5
Hyp coefficient = (Collagen weight) / (Hyp weight) = 90945.5/11458.1 = 7.94

Amino Acid	MW (g/mol)	Weight (g)	Number of residues (mol/1000 residues)
Hypro	131.1	11458.1	87.4
Asp	133.1	6455.4	48.5
Thr	119.1	2393.9	20.1
Ser	105.1	4193.5	39.9
Glu	147.1	10591.2	72
Pro	115.1	13052.3	113.4
Gly	75.07	25861.6	344.5
Ala	89.09	9033.7	101.4
Cys	121.1	302.8	2.5
Val	117.2	2496.4	21.3
Met	149.2	1387.6	9.3
Ile	131.2	1613.8	12.3
Leu	131.2	3227.5	24.6
Tyr	181.2	489.2	2.7
Phe	165.2	1833.7	11.1
Hylys	162.2	875.9	5.4
Lys	146.2	4064.4	27.8
His	155.2	869.1	5.6
Arg	174.2	8727.4	50.1
Total		108927.5	999.9

Table S2. Dry weights and total amino acids contents of murine pancreas

The dry weight of murine pancreas was measured after removal of fat by hexane treatment. The total amino acids content of whole murine pancreas was measured with a L-8800 amino acid analyzer after hydrolyzed with 6M HCl. Coefficient for the conversion of total amino acids content to tissue weight was calculated using a numerical formula as described below.

Coefficient = (Dry weight) / (Total amino acids content) = 62.1/37.9 = 1.64

	A) Pancreas weight (mg)	B) Fat (mg)	C) Dry weight (mg) (=A-B)	D) Total Amino Acid (mg)	E) Coefficient (=C/D)
No. 1	81.8	25.9	55.9	34.8	1.61
No. 2	102.7	39.7	63.0	37.0	1.70
No. 3	113.0	46.9	66.1	41.3	1.60
No. 4	142.1	78.7	63.5	38.5	1.65
Average	109.9	47.8	62.1	37.9	1.64

Supplementary information (full-length gels for Figs. 1C, 1E, 3D and 3E)

Fig. 1C

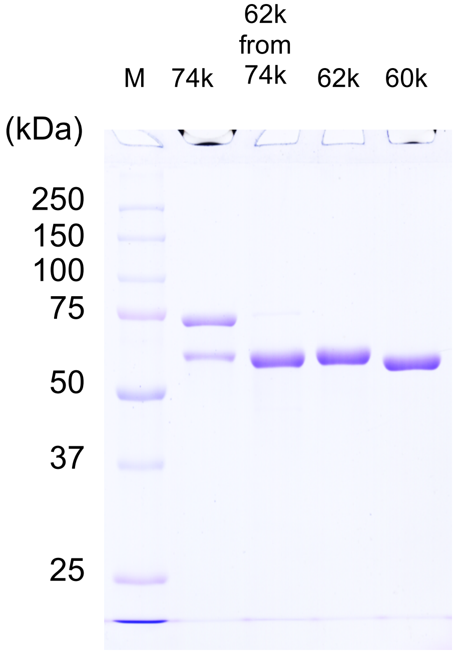

Fig. 1E

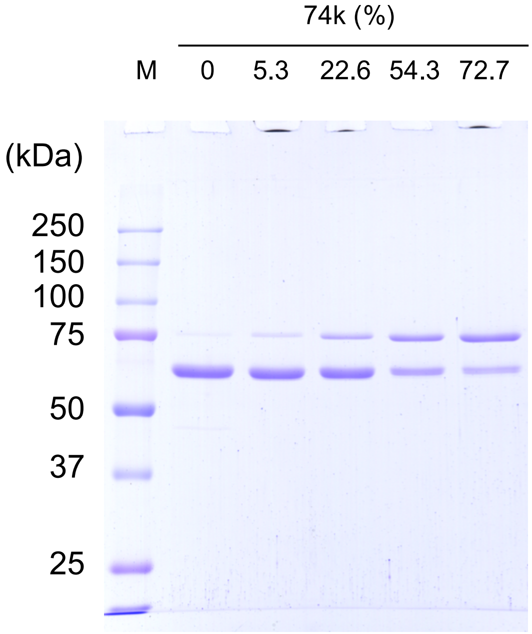

Fig. 3D

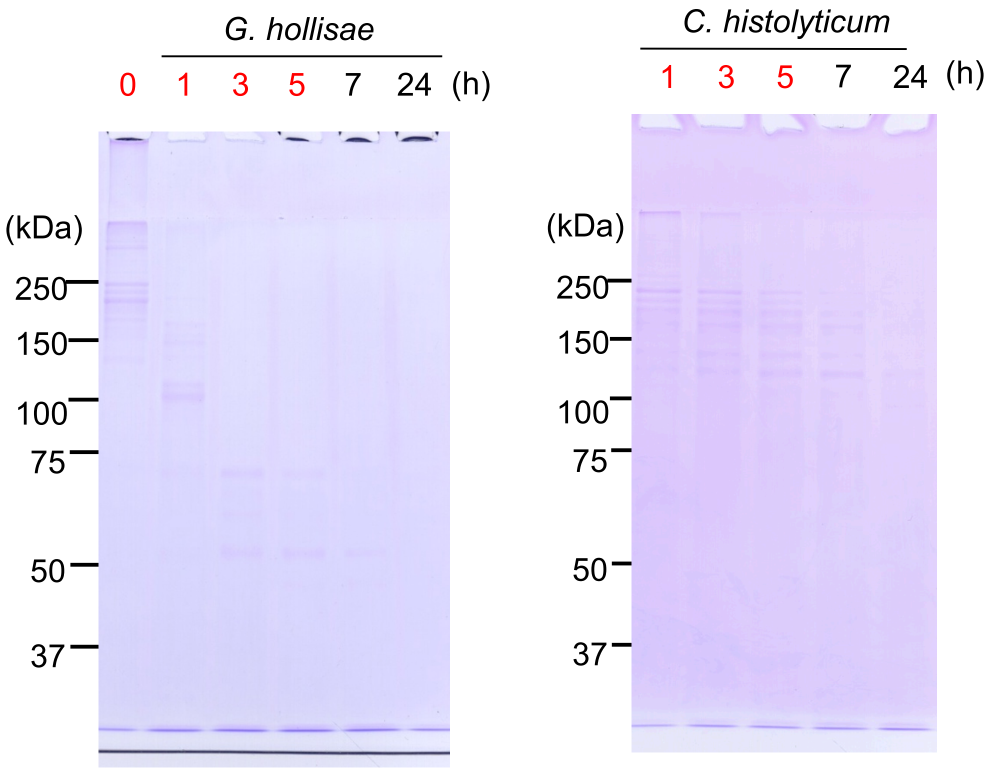
G. hollisae
0 1 3 5 7 24 (h)
(kDa)
250
150
100
75
50
37
C. histolyticum
1 3 5 7 24 (h)
(kDa)
250
150
100
75
50
37

Fig. 3E

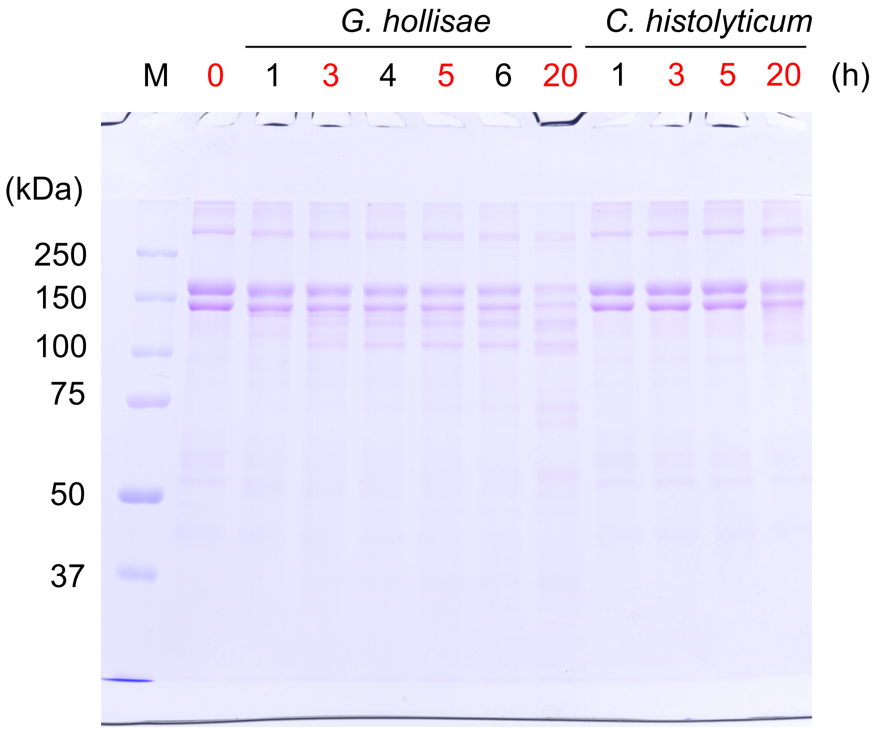
G. hollisae
C. histolyticum
M 0 1 3 4 5 6 20 1 3 5 20 (h)
(kDa)
250
150
100
75
50
37
